# Building Social Networks for Maternal and Newborn Health in Poor Urban Settlements: A Cross-Sectional Study in Bangladesh

**DOI:** 10.1371/journal.pone.0123817

**Published:** 2015-04-24

**Authors:** Alayne M. Adams, Herfina Y. Nababan, S. M. Manzoor Ahmed Hanifi

**Affiliations:** Centre for Equity and Health Systems, International Centre for Diarrhoeal Disease Research, Bangladesh (icddr,b). Dhaka, Bangladesh; Örebro University, SWEDEN

## Abstract

**Background:**

The beneficial influence of social networks on health and wellbeing is well-established. In poor urban settlements in Bangladesh, BRAC’s Manoshi programme trains community health workers (CHWs) to support women through pregnancy, delivery and postpartum periods. This paper test the hypothesis that the introduction of CHWs as weak ties into the social networks of Manoshi members mediates improvements in maternal and neonatal health (MNH) best practices by providing support, facilitating ideational change, connecting mother to resources, and strengthening or countering the influence of strong ties.

**Methods:**

1000 women who had given birth in the last three months were identified and interviewed as part of ongoing monitoring of 5 poor urban settlements in Dhaka, Bangladesh. A social networks questionnaire was administered which elicited women’s perceived networks around pregnancy, delivery and post-partum periods. Mediation analysis was performed to test the hypothesis that penetration of Manoshi CHWs into women’s perceived networks has a beneficial effect on MNH best practises.

**Results:**

The presence and influence of Manoshi CHWs in women’s networks significantly mediated the effect of Manoshi membership on MNH best practices. Respondents who were Manoshi members and who listed Manoshi CHWs as part of their support networks were significantly more likely to deliver with a trained birth attendant (OR 3.61; 95%CI 2.36–5.51), to use postnatal care (OR 3.09; 95%CI 1.83–5.22), and to give colostrum to their newborn (OR 7.51; 95%CI 3.51–16.05).

**Conclusion:**

Manoshi has succeeded in penetrating the perceived pregnancy, delivery and post-partum networks of poor urban women through the introduction of trained CHWs. Study findings demonstrate the benefits of moving beyond urban health care delivery models that concentrate on the provision of clinical services by medical providers, to an approach that nurtures the power of social networks as a means to support the poorest and most marginalized in changing behaviour and effectively accessing appropriate maternal services.

## Introduction

### Social Networks and Health

Strong epidemiological evidence suggests that individuals with diversified social networks who interact with family members, friends, neighbors and fellow workers, are married, or belong to social and religious groups, live longer and healthier lives than those who are less socially embedded and involved [1–4]. Social networks constitute the structural dimension of social relationships [1] and are typically appraised based on their dyadic characteristics (reciprocity, intensity/strength, formality and complexity) or physical features (size, homogeneity, geographic dispersion and density) [5, 6].

Several behavioral mechanisms have been proposed through which social networks affect health, including the provision of social support, social influence, social engagement, and access to resources [4, 6]. Social support represents the qualitative and behavioral aspects of social relations, and is commonly categorized into several subtypes, each of which has important consequences for health: emotional (love, care, sympathy and understanding); instrumental (money, labour or resources to aid with tangible needs); appraisal (support in decision-making); and informational (advice with respect to a particular need) [1, 6]. Social networks also affect health by acting as a conduit for social learning and social influence, through which existing norms are reinforced and/or new norms are diffused [7, 8]. Individuals who are highly interconnected and centrally located within local social networks are more likely to hear about innovations earlier and to have more opportunity for social comparison and influence [9].

Research also shows that particular features of social networks are linked to health behaviour and health status. For example, measures of reciprocity and intensity of network relationships are associated with positive mental health, while indicators of network density and proximity enhance health through their influence on social identity and the exchange of affective support [5]. By contrast, during times of transition and change, networks that are larger, more diffuse, and composed of less intense ties may be more adaptive because they are better at facilitating social outreach and exchanging new information [10]. Granovetter argues that less intense ties (weak ties) connect individuals who are significantly different from one another and therefore provide a bridge over which innovations or ideas can penetrate and diffuse, even though the decision making is influenced mainly by strong-tie networks [10].

However, not all social relationships are beneficial, nor do all types of networks furnish the same types of support. While most social contacts are positive and the provision of support is usually intended to be helpful, some social relationships are unwanted and adverse in nature—particularly for women [11]. The emotional or instrumental tension provoked by these types of social contacts is called relational strain and more often than not is inflicted by family members [1].

It is further noted that even though intimate social ties may be exceptional in furnishing some forms of social support, they are often subject to the same stressors, thus impeding the quality of support they provide, or provoking negative responses when their support is not well received [12]. While the role of professional helpers is to assist in these instances, and furnish information and resources that are not otherwise available in the social network, their empathy and commitment over the long term may be less than family members [5]. The recruitment and training of lay health professionals who are members of the communities they serve is one strategy that has proven effective in overcoming these limitations. These local health workers have a greater capacity for empathy due to shared life experiences, and are better able to foster supportive linkages within and beyond local networks [13].

### The Bangladesh Urban Context

Rapid urbanization in the Global South is accompanied by substantial social and structural challenges ranging from socio-economic inequities, to changing family arrangements and lifestyles. Largely a consequence of rural in-migration provoked by climate change, natural disaster, and economic pull factors [14], urban growth in Bangladesh is escalating at rate of 2.4% per year [15]. According to recent estimates, by mid-century, the total population of Bangladesh will reach an estimated 200 million, over 50% of which will live in urban areas, up from 34% in 2014 [15]. Most of this growth will concentrate in poor urban settlements that already comprise one third of the urban population. Urban health-related infrastructure and services are increasingly overwhelmed and health risks are exacerbated due to lack of access to clean drinking water, inadequate waste removal, insecure housing, and unsafe working conditions [16]. These risks are particularly concentrated in poor urban settlements where quality healthcare services are least accessible [17].

Maternal health is of particular concern, with only half of women living in poor urban settlements receiving ANC from a medically trained health worker [18]. Higher rates of home delivery by untrained birth attendants are also reported compared to women living in non-poor areas, with attendant risks of obstetric complications and delays in receiving emergency care [19]. According to the 2013 Urban Health Survey, only 37% of women living in poor urban settlements delivered in facilities compared to 65% of women resident in non-poor areas [18].

Bangladesh has a long history of investment in community-based delivery strategies that rely on social networks to extend primary health care to the poor and disadvantaged. With regards to family planning in particular, a social networks approach to family planning communication that involved specific targeting of opinion leaders by outreach workers was found to significantly predict ideational change around fertility and the adoption of modern contraception [9]. Other studies have also noted the role of outreach workers as change agents in terms of women’s fertility choices [20], and the positive impact of community-based peer counsellors on breastfeeding practises in Bangladesh [21].

The effectiveness of a social network approach in delivering health services in urban areas in Bangladesh is less established. Not only are linkages to formal service provision extremely limited with needs rapidly outpacing availability of care, the steady stream of rural migrants into poor urban settlements, and the destabilizing effects of tenancy insecurity and eviction, challenge service delivery structures and the social support networks on which many poor residents depend. For recent migrants, especially newly married women experiencing their first pregnancy, this context can be daunting and necessitate greater reliance on non-familial or less intimate networks in supporting pregnancy and navigating a complex urban health system. At the same time, social transitions of this nature are opportunities for change whereby conservative beliefs around maternal-neonate practices such as home delivery by untrained traditional birth attendants or pre-lacteal feeding, can be overcome. In this respect, nurturing networks of support around mothers living in poor urban settings that promote safe pregnancy, delivery and post-partum care may represent a timely and promising intervention.

### Maternal and Neonatal Health (MNH) Service Delivery: The BRAC Manoshi Model

In this context, a number of initiatives have been undertaken to support the MNH needs of poor urban women in Bangladesh, the most ambitious of which is Manoshi, a MNH programme run by BRAC, a historically rural-focused Non-Governmental Organization (NGO), which is now taking up the health and development challenges of urbanization. Initiated in 2007, Manoshi works in seven city corporations and 15 metropolitan areas in Bangladesh in the areas of: 1) capacity development of Community Health Workers (CHWs) and birth attendants; 2) health service provision for pregnant and lactating women, newborn and under-five children; 3) timely referral to quality health facilities; 4) community empowerment through development of women’s groups, and 5) linkage with government, local government, community people and NGOs [22].

An important feature of Manoshi is pregnancy identification, registration and follow-up regardless of where women seek care. Enrolment in Manoshi largely occurs through active identification of eligible members by CHWs (meeting the low socio-economic criteria defined by the programme), although spontaneous enrolment from expectant mothers is also possible. When eligible mothers refuse to be enrolled they are not considered Manoshi members. Once identified, pregnant women are encouraged to give birth in Manoshi delivery centers to assure privacy and hygienic delivery. In complicated cases, women are referred to pre-selected referral sites (both public and private) that offer comprehensive emergency obstetric care [22]. Frontline services are provided by *Shasthya Sebikas (SSs)* who are local women from the poor urban communities they are serving. SSs receive a three week training, and a small allowance to cover costs associated with monthly refresher training. Although unsalaried, SSs can access interest-free loans from BRAC, and a small income generated through the sale of basic health commodities. Responsible for 200 households each, SSs identify and register pregnant women, and through regular home visits, give information on danger signs of obstetric complications, and promote maternal and essential newborn care [22]. Supervision is provided by *Shasthya Kormis (SKs)*, who play a central role in referrals, and offer support to *Urban Birth Attendants* (UBAs) and Manoshi midwives responsible for clean and safe delivery at delivery centres located within poor urban settlements [22]. Both *SSs* and *SKs* are referred hereafter as community health workers (CHWs).

Over a 5-year period, baseline and endline sample surveys undertaken by an independent research institution (icddr,b) showed a rise in facility deliveries in Manoshi programme areas in Dhaka from 15% in 2007 to 59% in 2011, excluding those that occurred in Manoshi’s delivery centres (23% of total deliveries) [23]. The percentage of women receiving 4+ Antenatal Care (ANC) sessions has also risen from 27% in 2006 to 52% in 2011, and there are indications of a decline in rates of maternal and newborn death since programme inception [23].

For people with limited resources like those living within poor urban settlements, possessing a strong network may be critical to livelihood and coping with life’s problems or difficulties [24]. While formative research in Dhaka city has suggested that many poor urban residents report strong social and economic support networks, they are perceived to be less effective when coping with health-related problems [25]. By employing CHWs who support women around pregnancy and delivery, and nurturing linkages with community leaders and emergency obstetric services, Manoshi creates networks that encourage improved MNH practises and outcomes.

### Study objectives

This paper explores how the BRAC Manoshi programme addresses the challenges of maternal care and support in poor urban settlements. More specifically, it tests the hypothesis that women’s membership in Manoshi allows the introduction of CHWs as weak ties into women’s social network which function to mediate improvements in MNH behaviour by: providing support, facilitating ideational change, connecting the mother to resources, and strengthening/ countering the influence of strong ties. The paper also considers the structure and composition of women’s social networks in poor urban settlements, and identifies attributes that predict the adoption of positive MNH practices. Results from this analysis may have important implications for the design of urban MNH strategies and their evaluation, and provide evidence-based support to wider investment in interventions that nurture and extend social networks around a women’s maternity experience.

## Methods

### Study design

This cross-sectional study was conducted in conjunction with the International Centre for Diarhoeal Disease Research, Bangladesh (icddr,b)’s independent on-going monitoring of key MNH indicators under the Community-based Maternal, Newborn, and Child Health Programme (Manoshi) for Urban Bangladesh study. Monitoring occurred in five slum catchment areas in Dhaka City (Korail Bosti, Mohammadpur, Kamrangirchar, Sabujbag, and Mirpur) where Manoshi was operating over the period 2007–2012, with social network questions incorporated in monitoring activities from October to December 2011. Each poor urban settlement was visited in sequence, and every birth occurring in the last three months was registered. Based on this census sample, all mothers of surviving infants were interviewed, until a total sample size of 1000 was attained. Larger settlements (Kamrangirchar and Sabujbag) yielded larger samples of recently pregnant women, and no settlement was visited twice. Seven questionnaires were incomplete, resulting in a total sample size of 993 women: Korail Bosti (n = 99), Mohammadpur (n = 144), Kamrangirchar (n = 349), Sabujbag (n = 205), and Mirpur (n = 196).

Data were collected by icddr,b’s Manoshi monitoring team, all of whom were female and unknown to respondents. Supervised by two mid-level scientists at icddr,b, the team received classroom and field-based training on a short “social networks”module that was administered following routine monitoring questions. Data are available by request from icddr,b: http://www.icddrb.org/policies/cat_view/14445-policies.

The social networks questionnaire used in the study employed a simple ego-centric design in which the respondent (ego) elicited the names of the alters or nodes to whom she is directly connected. Reciprocity between ego and alters was not assessed, nor were indirect connections with other nodes captured within a bounded network [26]. Successfully applied in other low literacy populations [24], each respondent was asked to free list the names of those whom she perceives to support her around a specified domain, and to provide details about the characteristics of each alter, and the nature of the relationship. All responses were unprompted, so the list of network members was a function of ego’s perception and recall. Due to its simplicity, the advantage of this approach was the ability to gather data on a large sample size, thus enabling the application of classical statistical techniques to test hypotheses.

The questionnaire was comprised of three sections. The first section assessed the perceived availability of economic and emotional support by asking the respondent to identify those she considers “important” to her. The second section focused on networks of support around pregnancy, labour and delivery, and the post partum period. After having recorded the name of each alter, details were elicited about their age, sex, and residence, and the frequency and nature of support provided. Manoshi CHWs were indirectly identified in this manner. A third section requested additional information about the delivery itself: where it occurred, why and whether it was attended by a medically trained provider. Information was also collected on whether the respondent participated in any associations or group meetings in the last year, and related details such as the purpose of the group, its activities, and the number of meetings attended in the last three months.

### Measures

#### MNH variables

The dependent variables examined in the study were: 1) Utilization of a trained birth attendant; 2) Utilization of postnatal care, and; 3) Whether colostrum was fed to the newborn. A dichotomized variable was created for babies delivered under the supervision of a trained birth attendant, defined here as a trained health professional—i.e. a birth attendant, midwife, doctor or nurse—who is taught how to manage normal (uncomplicated) pregnancies, childbirth and the immediate postnatal period, and to identify, manage and refer complications in women and newborns [27].

A variable was included to represent postnatal care which is considered critical for the health and survival of both mother and newborn [28]. In this study, postnatal care refers to whether or not care was received within 24 hours of birth. Use of colostrum, a rich initial source of Immunoglobulin A (IgA) that is important for the survival of newborns, was similarly categorized [28].

#### Socio-demographic variables

Several socio-demographic variables were included in analysis given their known or likely impact on MNH and network measures. Membership in Manoshi was the primary independent variable which is hypothesised to improve MNH outcomes in this analysis. Manoshi membership was coded “yes” if the respondent was registered in the Manoshi record keeping system and receiving CHW visits. Other variables included membership in other organizations, respondents’ age, education, number of children, duration of living in the poor settlement and wealth quintile. Membership in other organizations was based on self-report. Respondents’ age was categorized based on median value; education and number of children were categorized using cut-offs that were conceptually sensible and which provided sufficient numbers for analysis, while duration of living in the poor urban settlement was coded as more or less than three years on the assumption that within three years most incoming residents would be sufficiently oriented to their new neighbourhood. Household wealth quintiles were constructed using principal component analysis by including all recorded household assets such as bed, radio, and bicycle [29], and dividing the sample into five categories, with higher quintiles corresponding to higher household wealth.

#### Social network variables

Explanatory variables were limited to network size and composition. A dichotomous variable representing network size was based on the median number of unique network members listed by respondents. Large networks were those with more than seven unique members, and small with less than seven members. The composition of networks was captured by information on the age, sex, relationship and proximity of each tie/network member identified by the respondent.

The presence and influence of particular ties i.e. husband, mothers or mothers-in-law and/or, CHWs, on MNH outcomes was also explored. For example, depending on whether a husband figured across any of the domains assessed in the study (economic, emotional, pregnancy, delivery, and postpartum), he would be coded “present” or “absent” in a respondent’s overall network. In this study, the presence of Manoshi CHWs (Shasthya Sebikas/SSs and Shasthya Kormis/SKs) was hypothesised to mediate the effect of Manoshi membership in improving MNH outcomes.

The percentage of network members who live in the same settlement was generated by dividing the number of network members who reside in the same settlement over total network size, and dichotomized on the basis of the median value. A separate variable representing the strength of emotional networks was derived based on the reported frequency of interaction, whereby everyday denotes frequent interaction, and anything less was considered regular/occasional.

### Ethical considerations

The ongoing monitoring system through which this study was fielded was approved by icddr,b’s Ethical Review Committee. Written informed consent was sought from all respondents’ prior to interview.

## Statistical analysis

Data analysis for this paper involved the assessment of simple frequency distributions and bivariate analysis exploring associations between Manoshi MNH indicators and network characteristics, as well as mediation analysis and multivariate logistic regression.

**Fig 1:**
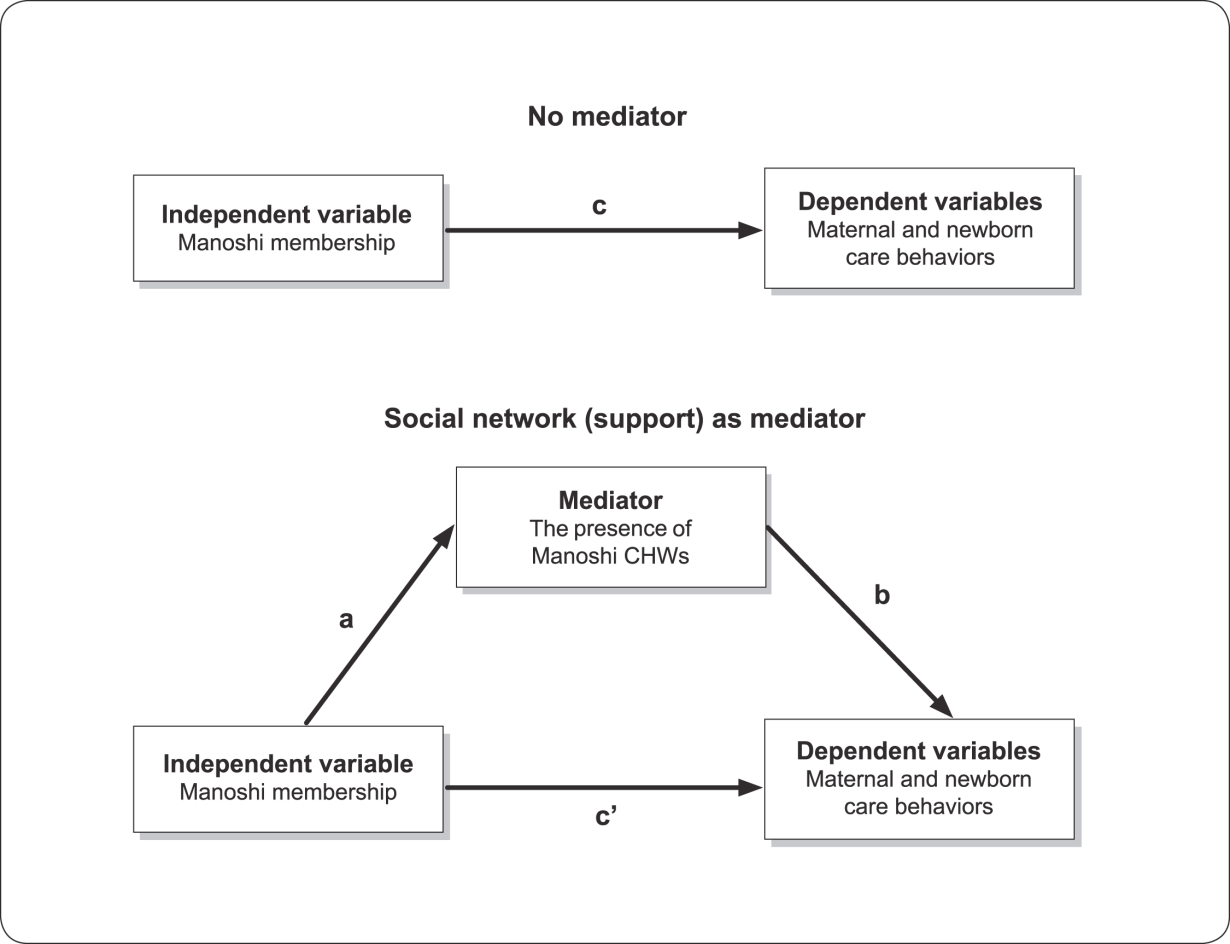
Conceptual model: the mediation effect of the presence of Manoshi CHWs on maternal and newborn care behaviour.

The potential mediation effect of the presence of Manoshi CHWs as a result of Manoshi membership was assessed using the criteria of Baron and Kenny [30]. Fig 1 depicts the hypothesised mediation model used to direct the analysis. Three conditions should be met to satisfy the criteria for mediation: (1) the association between the primary independent variable (Manoshi membership) and the potential mediator (the presence of Manoshi CHWs) must be significant (a ≠ 0 in the figure); (2) the association of the primary independent variable (Manoshi membership) with dependent variables (MNH behaviours) must be significant (c ≠ 0 in the figure), and (3) the mediator (the presence of Manoshi CHWs) must be significantly associated with the dependent variable controlling for the primary independent variable (Manoshi membership) (b ≠ 0 in the figure). Mediation is considered to have taken place if these criteria are satisfied and the association between the primary independent variable and the dependent variable is significantly reduced with the inclusion of the potential mediator in the model (c’ is statistically smaller than c). The significance of the presence of Manoshi CHWs’ mediation effect was assessed using the Sobel test [31].

The third step of regression in mediation analysis also permits an assessment of the social network related determinants of MNH best practises. In regression models, study site (Korail Bosti, Mohammadpur, Kamrangirchar, Sabujbag, and Mirpur) was included as an independent variable to adjust for potential clustering effects. Cluster correlated robust estimates of variance were employed to account for clustering within sites. The significant value for the results was set at p<0.05. All analysis was conducted using STATA version 11 (STATA Corp.).

## Results

### Respondent characteristics

Manoshi members and non-members differed significantly on a number of socio-demographic indicators at bivariate level. Manoshi members were younger, poorer, and more likely to have 1–5 years of education than non-members (Table 1). Significantly lower levels of membership in any organization were reported by Manoshi members, however perceived network size and the percentage of network members living in the same settlement where they were residing were significantly greater than non-members.

**Table 1:** Characteristics of respondents.

	Manoshi membership		
	No	Yes	P-value
	Freq (%)	Freq (%)	
**Age**			
≤ 21 yrs old	142 (47.65)	379 (54.53)	0.047
> 21 yrs old	156 (52.35)	316 (45.47)	
**Education**			
No education	49 (16.44)	65 (9.35)	0.001
1–5 yrs	169 (56.71)	469 (67.48)	
> 5 yrs	80 (26.85)	161 (23.17)	
**Parity**			
1	164 (55.03)	418 (60.14)	0.095
2	91 (30.54)	208 (29.93)	
≥3	43 (14.43)	69 (9.93)	
**Years in poor settlement**			
≤ 3 yrs	79 (26.51)	172 (24.75)	0.558
> 3 yrs	219 (73.49)	523 (75.25)	
**Household wealth quintiles**			
Poorest	53 (17.79)	153 (22.01)	0.026
Poor	69 (23.15)	155 (22.30)	
Middle	59 (19.80)	146 (21.01)	
Rich	48 (16.11)	136 (19.57)	
Richest	69 (23.15)	105 (15.11)	
**Membership in any organization**			
No	207 (69.46)	581 (83.60)	0.001
Yes	91 (30.54)	114 (16.40)	
**Network size**			
≤ 7	173 (58.05)	349 (50.22)	0.023
> 7	125 (41.95)	346 (49.78)	
**% of network members living in same poor settlement**			
≤ 90%	126 (42.28)	197 (28.35)	0.001
> 90%	172 (57.72)	498 (71.65)	
**Number of emotional network members**			
1	139 (46.64)	258 (37.12)	0.018
2	141 (47.32)	392 (56.40)	
3	18 (6.04)	45 (6.47)	
**Total respondents**	298	695	

### Network size and composition

Among the five domains around which respondent networks were elicited, the largest was related to labour and delivery. When individuals appearing in more than one network were removed, the average total network size was seven members in both Manoshi member and non-member groups. Over 80% of network members listed by women in the study lived in close proximity, with only a small percentage indicating post partum and delivery support systems beyond the poor settlement in which they resided (8%), or outside of Dhaka (3%) (Table not shown).

Table 2 presents the composition of each network domain in terms of who is providing support, defined here as the relationship between each network member and the woman respondent. Results emphasize the primacy of husbands, mother/in-laws and other family members in women’s post-partum networks, and the role of family members and husbands in economic networks. Emotional networks were largely comprised of family and friends, followed by husbands and Manoshi CHWs. Manoshi CHWs were the most frequently listed network member in women’s pregnancy networks in both member and non-member groups. Perceived networks around labour and delivery were largely comprised of Manoshi health workers (UBAs/midwives) and other health workers, with Manoshi members relying proportionately more on Manoshi health workers than non-members.

**Table 2:** Composition of network members (%) by Manoshi membership in five network domains.

		% of total network members							
Network	Manoshi		Mother/				Manoshi	Manoshi	Non-BRAC
Domain	Membership	Husband	in-law	Family	Friends	Landlord	CHWs	UBAs/	Health
								Midwives	Worker
**Economic**	**No**	27.88	7.51	30.38	18.36	15.86	0	0	0
	**Yes**	27.63	5.16	32.79	21.05	13.37	0	0	0
**Emotional**	**No**	14.85	10.23	41.58	24.75	1.32	6.60	0.00	0.66
	**Yes**	13.84	7.87	40.30	26.87	0.95	9.91	0.27	0.00
**Pregnancy**	**No**	2.75	3.03	5.51	1.65	0	69.15	1.93	15.98
	**Yes**	1.53	2.20	2.01	0.86	0	85.76	2.01	5.64
**Delivery**	**No**	7.19	11.80	14.10	10.94	0	4.17	16.12	35.68
	**Yes**	7.48	8.93	6.79	5.34	0.12	10.27	34.16	26.91
**Postpartum**	**No**	34.38	40.63	22.92	1.04	0	0.00	0.00	1.04
** **	**Yes**	36.48	30.33	28.69	3.28	0	0.41	0.41	0.41

### Kinds of support provided

Across all networks, the most prevalent form of assistance provided to mothers was instrumental support (62%), followed by information/appraisal support (27%) and emotional support (11%) (Table not shown). During pregnancy, informational support (58%) was the most frequently mentioned help received from network members, followed by instrumental support (29%) and emotional support (13%). For labour and delivery, instrumental support was the most important, especially in the form of care (19%), assistance with the delivery process (15%), and getting to the hospital (13%). During the postpartum period, help with housework (42%) and care for mother and child (39%) accounted for the bulk of instrumental support provided by network members.

Table 3 presents the kinds of support provided by different network members during pregnancy, delivery and post-partum periods. On average, CHWs were the most important provider of overall support in terms of total numbers, representing the main conduit of information and a noted source of emotional support and help in getting to the hospital. Differences in patterns of support comparing Manoshi members and non-members are largely explained by the relative contribution of CHWs. For Manoshi members, CHWs provided the main source of informational, instrumental, and emotional support during pregnancy, delivery and post-partum periods. Among non-members, most perceived instrumental and emotional support came from family and mothers/in-laws, although Manoshi CHWs were considered the main source of information in both groups. As expected, Manoshi health workers and other health workers represented the principle source of help with delivery.

**Table 3:** Distribution of support (%) provided by network members during pregnancy, delivery and postpartum periods.

	Manoshi	% of total support									Total
Relationship	membership	Informational	Instrumental							Emotional	support
			Money	Housework	Care	Delivery	Accompany*	Medicine	Total		
**Husband**	**No**	9.33	70.23	19.28	16.22	0.17	30.11	39.89	21.34	16.65	17.78
	**Yes**	7.09	76.80	22.46	19.82	0.07	26.17	58.16	16.76	16.76	18.22
**Mother/in-laws**	**No**	12.06	12.94	39.05	25.79	8.77	18.43	4.26	25.04	21.3	21.34
	**Yes**	6.42	7.53	31.89	19.16	4.25	14.06	2.37	15.14	15.14	14.97
**Family**	**No**	16.8	9.06	35.51	25.79	10.29	25.18	3.72	24.67	20.69	22.23
	**Yes**	12.24	9.83	36.84	21.72	4.18	21.68	2.63	16.46	16.46	18.88
**Friend**	**No**	8.24	1.62	5.95	6.14	12.82	5.29	1.06	6.33	5.14	6.68
	**Yes**	5.11	1.38	8.14	5.76	5.45	7.88	1.05	4.02	4.02	5.69
**Landlord**	**No**	0.33	0	0	0.06	0	0	0	0.02	0.12	0.11
	**Yes**	0.22	0.31	0.19	0.13	0.14	0.07	0.53	0.05	0.05	0.17
**Manoshi CHW**	**No**	32.9	5.5	0	6.14	4.89	20.44	1.6	5.57	17.38	13.81
	**Yes**	49.01	3.84	0.32	10.56	10.26	28.78	3.68	26.7	26.7	22.49
**Manoshi health worker**	**No**	3	0.32	0.07	6.53	18.72	0.18	3.72	4.83	6.12	4.51
	**Yes**	9.66	0.15	0.16	14.41	42.75	0.78	3.68	11.76	11.76	10.66
**Non-BRAC health**	**No**	17.35	0.32	0.14	13.32	44.35	0.36	45.74	12.18	12.61	13.54
**worker**	**Yes**	10.25	0.15	0	8.45	32.91	0.59	27.89	9.11	9.11	8.93

* Assistance getting to the facility

### Social network related determinants of MNH best practices

Table 4 column B displays the results of multivariate analysis that considered the relative importance of social network measures and other explanatory factors in predicting three MNH best practices: use of a trained birth attendant for delivery, postnatal care and giving colostrum as the first food to the newborn. The presence or absence of certain network ties in a woman’s perceived network exerts a significant effect on the adoption of positive MNH practices. Women who listed CHWs in their network were two times (OR 2.13; 95%CI 1.40–3.25) more likely to deliver with a trained birth attendant, while the presence of husbands increased the odds by a factor of four (OR 4.36; 95% CI 2.85–6.67). When mothers/in-laws or friends figured in a woman’s perceived network, there was a significant negative effect on the probability of delivering with a trained birth attendant (OR 0.48; 95% CI 0.31–0.74 and OR 0.16; 95% CI 0.10–0.25 respectively). A negative effect on delivery with trained birth attendant was also observed among women whose networks were largely concentrated within the urban settlement where they reside (OR: 0.66; 95% CI 0.43–1.00).

**Table 4:** Multiple logistic regression exploring the mediation effect of Manoshi CHWs in explaining the presence of Manoshi membership on MNH best practices.

	Trained birth attendant at delivery			Postnatal care received			Colostrum given to newborn		
		OR (95%CI)			OR (95%CI)			OR (95%CI)	
	% Use	A	B	% Use	A	B	% Use	A	B
**Maternal Age**									
≤ 21 yrs old	79.46	1.00	1.00	87.33	1.00	1.00	93.28	1.00	1.00
> 21 yrs old	83.05	1.06 (0.53–2.14)	1.03 (0.51–2.06)	91.74	1.34 (0.49–3.67)	1.32 (0.46–3.81)	95.97	0.92 (0.30–2.79)	0.91 (0.27–3.07)
**Parity**									
1	80.24	1.00	1.00	88.83	1.00	1.00	93.47	1.00	1.00
2	80.60	1.08 (0.53–2.20)	1.19 (0.59–2.40)	89.97	0.74 (0.27–1.98)	0.85 (0.30–2.40)	96.66	2.37 (0.74–7.61)	2.53 (0.71–9.05)
≥3	87.5	2.34 (0.89–6.20)	2.44 (0.94–6.32)	91.07	0.84 (0.22–3.21)	0.80 (0.20–3.19)	94.64	2.24 (0.54–9.21)	1.90 (0.41–8.76)
**Years in poor settlement**									
≤ 3 yrs	73.71	1.00	1.00	79.68	1.00	1.00	89.64	1.00	1.00
> 3 yrs	83.69	1.45 (0.92–2.27)	1.34 (0.85–2.10)	92.72	2.33 (1.33–4.07)*	1.96 (1.10–3.49)*	96.23	1.78 (0.90–3.53)	1.53 (0.77–3.03)
**Maternal Education**									
No education	72.81	1.00	1.00	86.84	1.00	1.00	89.47	1.00	1.00
1–5 yrs	83.23	1.61 (0.85–3.04)	1.77 (0.93–3.35)	89.97	0.88 (0.41–1.87)	1.04 (0.47–2.31)	94.83	1.90 (0.81–4.50)	1.92 (0.76–4.87)
> 5 yrs	79.67	1.26 (0.64–2.50)	1.46 (0.73–2.89)	89.21	0.64 (0.27–1.49)	0.85 (0.35–2.05)	96.27	2.17 (0.73–6.42)	2.46 (0.79–7.62)
**Household wealth quintiles**									
Poorest	74.27	1.00	1.00	79.13	1.00	1.00	87.86	1.00	1.00
Poor	79.91	1.4 (0.82–2.39)	1.44 (0.85–2.44)	87.05	2.15 (1.15–4.01)*	2.47 (1.31–4.66)*	93.75	2.69 (1.13–6.38)*	2.84 (1.16–6.93)*
Middle	83.41	1.37 (0.75–2.53)	1.35 (0.74–2.48)	92.68	3.26 (1.48–7.17)*	3.51 (1.64–7.53)*	96.10	3.35 (1.20–9.37)*	3.18 (1.17–8.67)*
Rich	85.33	1.53 (0.79–2.96)	1.42 (0.73–2.78)	94.02	2.98 (1.29–6.90)*	2.73 (1.10–6.77)*	98.37	7.10 (2.06–24.52)*	6.66 (1.81–24.55)*
Richest	83.91	1.96 (1.03–3.73)*	1.82 (0.96–3.45)	95.98	7.80 (2.69–22.62)*	6.79 (2.33–19.80)*	97.70	6.75 (1.77–25.75)*	5.64 (1.48–21.42)*
**Manoshi membership**									
No	66.11	1.00	1.00	79.53	1.00	1.00	86.24	1.00	1.00
Yes	87.63	4.25 (2.82–6.39)*	3.61 (2.36–5.51)*	93.67	4.18 (2.50–6.99)*	3.09 (1.83–5.22)*	98.13	9.79 (4.56–21.02)*	7.51 (3.51–16.05)*
**Network size**									
≤ 7	77.2	1.00	1.00	87.16	1.00	1.00	93.30	1.00	1.00
> 7	85.56	1.4 (0.93–2.10)	1.15 (0.76–1.74)	91.93	1.31 (0.77–2.24)	0.86 (0.50–1.50)	95.97	1.15 (0.61–2.16)	0.85 (0.43–1.68)
**Presence of network member**									
**1. Husband**									
Absent	60.56	1.00	1.00	76.06	1.00	1.00	90.14	1.00	1.00
Present	82.75	4.81 (3.17–7.32)*	4.36 (2.85–6.67)*	90.46	5.41 (3.32–8.81)*	5.06 (3.06–8.38)*	94.90	3.59 (1.76–7.34)*	3.38 (1.61–7.08)*
**2. Mother/ in-laws**									
Absent	85.77	1.00	1.00	92.89	1.00	1.00	94.76	—	—
Present	76.39	0.44 (0.29–0.68)*	0.48 (0.31–0.74)*	85.83	0.46 (0.26–0.81)*	0.52 (0.29–0.95)*	94.35		
**3. Friends**									
Absent	88.83	1.00	1.00	94.41	1.00	1.00	96.93	1.00	1.00
Present	76.85	0.17 (0.11–0.27)*	0.16 (0.10–0.25)*	86.61	0.24 (0.14–0.42)*	0.20 (0.11–0.37)*	93.23	0.34 (0.17–0.70)*	0.31 (0.15–0.65)*
**4. Manoshi CHWs**									
Absent	60.94	—	1.00	67.97	—	1.00	79.69	—	1.00
Present	84.16		2.13 (1.40–3.25)*	92.60		5.19 (3.03–8.91)*	96.76		2.89 (1.35–6.20)*
**% of network members living in same poor settlement**									
≤ 90%	85.07	1.00	1.00	89.47	—	—	95.68	1.00	1.00
> 90%	76.2	0.69 (0.93–2.10)	0.66 (0.43–1.00)	89.40			93.14	0.78 (0.38–1.60)	0.77 (0.37–1.61)
**Number of emotional network members**									
1	79.09	—	—	86.40	1.00	1.00	93.20	—	—
2	82.18			91.74	1.58 (0.94–2.66)	1.64 (0.96–2.79)	95.31		
3	85.71			88.89	0.70 (0.22–2.21)	0.54 (0.17–1.74)	96.83		

Model A = Without the presence of Manoshi CHWs

Model B = With the presence of Manoshi CHWs

* Significant result (p <0.05)

— Variable not included in the final model

As regards the probability of using postnatal care, a positive association was apparent with increasing duration of residence; respondents who had been living in the same settlement for more than three years were two times (OR 1.96; 95%CI 1.10–3.49) more likely to receive post natal care. Manoshi members were three times (OR 3.09; 95%CI 1.83–5.22) more likely to receive post-natal care than non members. The presence of a CHW or husband in a woman’s perceived network increased the use of postnatal care by five times (OR 5.19; 95%CI 3.03–8.91 and OR 5.06; 95% CI 3.06–8.38 respectively). Whereas mothers/in-laws (OR 0.52; 95% CI 0.29–0.95) or friends (OR 0.20; 95% CI 0.11–0.37) had a notable negative effect on this care practice.

Manoshi members were seven times (OR 7.51; 95%CI 3.51–16.05) more likely to give colostrum to their newborns compared to non-members. The probability of giving colostrum was approximately three times greater among women identifying Manoshi CHWs or husbands as network members (OR 2.89; 95%CI 1.35–6.20 and OR 3.38; 95% CI 1.61–7.08 respectively). The presence of friends in women’s perceived networks had the opposite effect, decreasing the probability of giving colostrum by 70% (OR: 0.31; 95% CI 0.15–0.65).

### The mediation effect of Manoshi CHWs in explaining the impact of Manoshi membership on MNH best practices

The extent to which the presence of CHWs in a woman’s network explained the positive impact of Manoshi membership on MNH best practices was tested using mediation analysis. Both bivariate analysis (not controlling for confounding variables) and multiple regression (controlling for confounding variables) demonstrated that Manoshi members consider Manoshi CHWs to be important social network members (Tables not shown), thereby satisfying the first criterion of mediation.

The second criterion of potential mediation is demonstrated in Table 4, regression model A. A significant association between Manoshi membership and each MNH best practice is revealed. Controlling for other confounders, Manoshi members were four times more like to deliver with the help of birth attendant, four times more likely to receive postnatal care, and almost ten times more likely to give colostrum to their newborns.

To test the last criterion for proof of mediation, the CHW variable was added to regression model B while controlling for Manoshi membership (Table 4). This model assessed whether the association of Manoshi membership with each outcome decreased compared to model A which does not include Manoshi CHWs. This last criterion was met for all outcomes: the presence of Manoshi CHWs was found to significantly predict delivery by a trained birth attendant, postnatal care use, and use of colostrum as the newborn’s first food of Manoshi members, and a reduction in the association of Manoshi membership with all three MNH best practices was noted when comparing models A and B.

To test the significance of the mediation effect of Manoshi CHWs in explaining the impact of Manoshi membership on key positive MNH health practices, the Sobel test was applied. A statistically significant mediation effect of Manoshi CHWs was demonstrated for all three MNH practices (p < 0.001) (see S1 Table).

## Discussion

Manoshi’s strategy to promote safe motherhood and newborn health in poor urban settlements relies on well-supervised systems of home-visiting and timely referral, supported by community engagement through the formation of women’s support groups and MNH committees [22]. An implicit theory of change undergirding programme strategy is the incorporation of local trained CHWs into women’s social networks to extend support around pregnancy and delivery, and timely linkage with maternity services. This study represents a first empirical investigation of this theorized mechanism of change, and whether it successfully predicts positive MNH health behaviours.

Results demonstrate that Manoshi CHWs have successfully penetrated the networks on which poor urban women rely during the critical reproductive phases of pregnancy, delivery and the post-partum period, and in so doing, have contributed toward improvements in maternal and newborn health behaviours. Women who free listed Manoshi CHWs as part of their network were two to five times more likely to adopt positive MNH practices. Mediation analysis further confirms the specific contribution of CHWs as network members to positive MNH practices, over and above membership in Manoshi. This finding is consistent with previous studies in Bangladesh that linked the use of outreach workers [9] and peer counselors [21] with increased modern contraceptive use and improved breastfeeding practice respectively.

Viewed in terms of social networks theory, the positive effects of CHWs as network members around pregnancy and delivery appear to endorse Granovetter’s (1973) notion of the strength of “weak ties”. Women whose networks extend beyond a closed circle of friends and family (weak ties) have a greater likelihood of being exposed to new ideas and applying positive behaviours in pregnancy and delivery. It is also interesting that in addition to their important role as a source of informational support, Manoshi CHWs are perceived to provide emotional support by many women respondents. This finding supports the recommendation of Heaney and Israel (2008) that employing trained health workers who are members of the communities in which they are working may help overcome the lack of empathy typical of professional health workers [5].

The effects of other social network features on MNH best practices were also assessed, including network size, the percentage of network members who live in the same settlement, and the strength of perceived emotional networks, however, none had a significant effect in multivariate analysis. The presence of “strong ties”, defined here as network members related to or close to ego, did predict MNH practices, but not always in an optimal direction. For example, the presence of husbands in a woman’s perceived pregnancy and delivery network had a positive association with MNH practices. The role of husbands in determining pregnancy outcomes is well-established in the literature, although the mechanisms of influence remain poorly understood; largely focusing on their contribution as a biological donor, financial provider, or source of unpredictable stress or support [32]. In poor urban settlements where nuclear family structures predominate, the importance of husbands’ in networks supporting women around the pregnancy and childbirth may be a function of the absence of proximate extended family on whom women can rely. Interestingly, there is a strong correlation between women reporting husbands in their network and those reporting the presence of CHWs. While causality cannot be ascertained, it is plausible that husbands, many of whom work long hours, encourage their wives to avail CHW support. Alternatively, the presence of CHWs in a women’s network may function to encourage male involvement.

Multivariate analysis also demonstrated the adverse effect of “strong” ties that are close in terms of kinship or intimacy. Women who report the presence of mothers/in-laws or friends in their networks are less likely to practise positive MNH behaviours. According to network theory, the influence of “strong” ties over individual behaviour is often very conservative [33], with decision-making tending to reflect traditional views and practices that may be harmful to expectant mothers and their infants. It is plausible that the conservative influence of Mothers/in-laws and friends work to deter women’s uptake of innovations around delivery and post-partum practices. Individuals with few weak ties that extend beyond the confines of proximate friends and family may be deprived of novel information from the wider social system, and hence more reliant on the provincial news and views of their close networks [34].

Findings from this study provide persuasive evidence that enhancing the support networks of poor urban women around pregnancy and childbirth has a beneficial impact on the use of best MNH practises. Equally interesting is evidence of diffusion effects beyond those who claim direct membership in the programme. Even among non-members, substantial numbers of women free list Manoshi CHWs in their pregnancy and delivery networks. Also remarkable is the magnitude of overall improvement in MNH best practises across the sample compared to previous programme evaluations [35]. Strong diffusion effects beyond direct programme members have been noted in other studies in Bangladesh [7–9], and suggest an untapped potential for behaviour change that interventions should more deliberately foster.

Several limitations of study design must also be noted. Social network data was limited to perceived networks and reciprocity was not assessed. Inconsistencies in the size of pregnancy and delivery networks may also have occurred related to recall bias: it far easier to list those providing assistance over the brief delivery period compared to 9 months of pregnancy. Finally, the cross-sectional nature of study design precludes discussion of causality. We cannot claim that women’s networks are responsible for better MNH practices as it is equally possible that women who employ those practices are inherently more able to nurture strong supportive networks.

## Conclusion

Irrespective of study limitations, results are sufficiently robust to conclude that Manoshi has succeeded in penetrating women’s perceived networks, and that CHWs successfully function as “weak ties” enabling the flow of new information and support around best MNH practices. In the context of rapid urban change characterized by the explosive growth of poor urban settlements and the paucity of available primary care services, Manoshi’s “social networks” approach demonstrates the health benefits of moving beyond static health care delivery models that concentrate on the provision of medical services by medical providers, to an approach that nurtures the power of social networks in supporting the poor and marginalized around healthy pregnancy, and timely access to MNH services. Financial and social barriers, including formal and informal service costs, and the conservative influence of older generations, also need to be taken into consideration in efforts to encourage MNH best practices in urban settings.

## Supporting Information

S1 Table Sobel test of the significant mediation effects of CHWs. (XLS)

S1 Fig Conceptual model: the mediation effect of health literacy on racial/ethnic and educational disparities in health and preventive health behaviours in older adults. (EPS)
